# Allosteric Modulation of αβδ GABA_A_ Receptors

**DOI:** 10.3390/ph3113461

**Published:** 2010-11-03

**Authors:** Hua-Jun Feng

**Affiliations:** McGovern Institute for Brain Research, Massachusetts Institute of Technology, Cambridge, MA 02139, USA; E-Mail: fenghj@mit.edu; Tel.: +1-617-324-0135; Fax: +1-617-324-6752.

**Keywords:** GABA_A_ receptors, δ subunit, modulation, kinetics

## Abstract

GABA_A_ receptors mediate the majority of the fast inhibition in the mature brain and play an important role in the pathogenesis of many neurological and psychiatric disorders. The αβδ GABA_A_ receptor localizes extra- or perisynaptically and mediates GABAergic tonic inhibition. Compared with synaptically localized αβγ receptors, αβδ receptors are more sensitive to GABA, display relatively slower desensitization and exhibit lower efficacy to GABA agonism. Interestingly, αβδ receptors can be positively modulated by a variety of structurally different compounds, even at saturating GABA concentrations. This review focuses on allosteric modulation of recombinant αβδ receptor currents and αβδ receptor-mediated tonic currents by anesthetics and ethanol. The possible mechanisms for the positive modulation of αβδ receptors by these compounds will also be discussed.

## 1. Introduction

GABAergic neurotransmission mediates the prevalent inhibition in the mature brain [1,2,3]. The neurotransmitter involves in this signaling is γ-aminobutyric acid (GABA), which is biosynthesized from glutamate by glutamate decarboxylase (GAD) and degraded by GABA transaminase (GABA-T) [3]. GABA is purported to act as a primary neurotransmitter by 30-40% of all CNS neurons [2,4]. GABA is released from vesicles at synapses in a Ca^2+^-dependent manner [3,5]. Also, a Ca^2+^-independent cytoplasmic GABA release occurs mainly at extrasynaptic site, which may be caused by reversal transport of GABA molecules by GABA transporters (GAT) [2,3]. Upon release, GABA activates ionotropic GABA_A_ and GABA_C_ receptors as well as metabotropic GABA_B_ receptors. Termination of GABA action in the synaptic cleft is achieved by diffusion and active reuptake by GAT localized on presynaptic nerve endings and astrocytes [3]. Four GAT subtypes have been identified [6], and neurons mainly express GAT-1 and GAT-2/3 [7].

GABA_A_ receptors mediate the majority of fast inhibition in the adult brain [1,2]. Activation of GABA_A_ receptors results in two types of GABAergic inhibition: phasic and tonic inhibition. While phasic inhibition, mediated by inhibitory postsynaptic currents (IPSCs), is produced by brief exposure of postsynaptic GABA_A_ receptors to high concentrations of GABA, tonic inhibition is generated by continuous activation of extrasynaptic GABA_A_ receptors by low ambient concentrations of GABA [8,9,10,11]. In many brain regions such as thalamus and hippocampus, GABA_A_ receptor-mediated currents are predominantly contributed by tonic currents, which account for ~75%-90% of total inhibitory currents [8,12,13]. Therefore, the tonic inhibition plays a major role in modulating neuronal excitability in these brain areas. 

**Figure 1:**
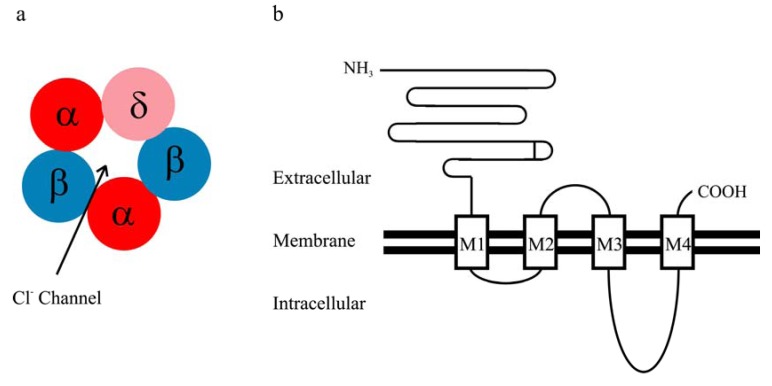
**(a)** The assumed stoichiometry of the αβδ receptor (α:β:δ = 2:2:1); **(b)** Schematic presentation of the topology of a GABA_A_ receptor subunit.

GABA_A_ receptors are heteropentameric chloride ion channels, and multiple GABA_A_ receptor subunit subtypes as well as splice variants have been identified, including α1-α6, β1-β3, γ1-γ3, δ, ε, π and θ [1]. Like the other members of the cys-loop receptor family, each GABA_A_ receptor subunit is composed of a long extracellular N terminus, four transmembrane domains (M1-M4), one extracellular M2-3 loop, two intracellular loops (M1-2 and M3-4) and a short extracellular C terminus (Figure 1). It has been reported that αbg and αbd receptors are the predominant isoforms present *in vivo* [14], primarily mediating phasic and tonic inhibition, respectively [9]. The α1β2γ2 isoform is the ubiquitous and predominant synaptic receptors in the brain [14,15]. On the other hand, the αbd GABA_A_ receptor is localized extra- or perisynaptically [9,10]. The δ subunit mainly co-assembles with the α6 subunit in the cerebellum [16,17] and with the α4 subunit in several brain regions such as thalamus and cortex [18,19,20,21]. An intimate association between δ and α1 subunits was observed in hippocampus [22], but was not detected in thalamus [19]. The α4βd receptor is the major δ subunit-containing GABA_A_ receptor in the brain [14]. 

## 2. Kinetic Properties of αβδ GABA_A_ Receptor Currents

**Figure 2:**
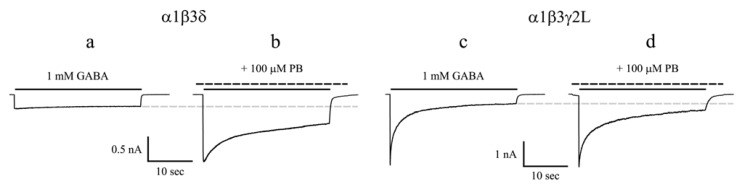
**(a)** The whole-cell current trace of α1β3δ receptors evoked by a saturating concentration of GABA displayed slow desensitization; **(b)** Pentobarbital substantially enhanced the peak and steady-state current amplitudes and increased the desensitization of α1β3δ receptors; **(c)** The whole-cell current trace of α1β3γ2L receptors evoked by a saturating concentration of GABA exhibited extensive and fast desensitization; **(d)** Pentobarbital did not potentiate the peak current amplitude but enhanced the steady-state current amplitude of α1β3γ2L receptors. Pentobarbital decreased the desensitization of α1β3γ2L receptors. The solid line above each current trace denotes the duration of GABA application (28 sec), and the dashed line denotes that of pentobarbital application. The gray dashed line indicates the level of steady-state current for GABA controls. PB, Pentobarbital. Modified from [26].

Whole cell currents evoked by saturating concentrations of GABA are always smaller for α1β3δ receptors than for α1β3γ2L receptors (Figure 2a,c) [23,24,25,26]. The extent of desensitization of αβδ receptor currents is dependent on the α subunit (Table 1). α4β2/3δ or α6β3δ currents evoked by saturating concentrations of GABA display considerable extent of desensitization [25,27,28,29]. However, compared with their counterpart α4β3γ2L and α6β3γ2L receptors [25,30], the desensitization of α4β3δ and α6β3δ receptors is relatively slower, lacking the fast component. α5β3δ receptors are poorly expressed in HEK293T cells, and the desensitization of this receptor isoform seems to be slower than that of α5β3γ2L receptors [29]. α1β2/3δ currents evoked by saturating concentrations of GABA exhibit very slow desensitization, some of which have minimal or no desensitization (Figure 2a) [23,26,31]. Structural investigations using δ-γ2L chimeras showed that the N terminus and two adjacent residues (V233, Y234) in M1 of the δ subunit contributed to the slow desensitization of α1β3δ receptors [32]. α1β3δ and α4β3δ currents deactivate faster than α1β3γ2L and α4β3γ2L currents, respectively [26,29]. The deactivation of α5β3δ and α6β3δ currents is slower than that of α1β3δ and α4β3δ currents and may not be different from their counterpart γ2L subunit-containing receptor currents (Table 1) [29]. 

**Table 1:** Comparison of GABA current kinetics among αβ3δ receptors with different α subunits. Adapted from [29].

	**α1**β3δ	**α4**β3δ	**α5**β3δ	**α6**β3δ
Desensitization	24.8 ± 6.5%	53.4 ± 2.1%	36.2 ± 4.4%	44.7 ± 3.9%
Deactivation	125.3 ± 10.5 ms	117.8 ± 13.5 ms	345.7 ± 87.4 ms	449.1 ± 80.9 ms

## 3. Modulation of αβδ Receptors by Anesthetics

### 3.1. Barbiturates

Barbiturates are widely used general anesthetics and exert their actions in the brain by interacting with GABA_A_ receptors [33]. Pentobarbital, the prototypic barbiturate commonly tested in laboratories, affects GABA_A_ receptor function in a concentration- and use-dependent manner. That is, at low concentrations, pentobarbital potentiates GABA_A_ receptor currents [26,34,35]. At higher concentrations, pentobarbital can directly activate GABA_A_ receptors [26,34,35,36]. At very high concentrations (mM), pentobarbital suppresses GABA_A_ receptor function via an open channel block mechanism [26,37,38,39]. These concentration- and use-dependent properties of barbiturates on GABA_A_ receptor function are also observed in other GABA_A_ receptor modulators [40,41]. 

Interestingly, chronic treatment with and subsequent withdrawal of pentobarbital in animals alter the expression of GABA_A_ receptor δ subunit in certain brain region [42], suggesting anesthetics may exert effects on αβδ receptors. Using a novel fluorescence resonance energy transfer-derived measurement of membrane potential, Adkins *et al.* reported that α4β3δ receptor response was markedly potentiated by pentobarbital [43]. Subsequent electrophysiological study showed that pentobarbital produced a greater potentiation of α4β3δ currents than α4β3γ2 currents evoked by sub-maximal concentrations of GABA [27]. We compared allosteric modulation by pentobarbital of α1β3δ and α1β3γ2L currents evoked by sub-maximal as well as saturating concentrations of GABA using a rapid drug application device. At a sub-maximal concentration of GABA (1 μM), pentobarbital at 100 μM enhanced peak current amplitude, increased the desensitization and prolonged the deactivation of α1β3δ and α1β3γ2L currents to a similar extent [26]. On the other hand, pentobarbital differentially modulated α1β3δ and α1β3γ2L currents evoked by a saturating concentration of GABA (1 mM). Pentobarbital substantially enhanced the peak current amplitude and increased the desensitization of α1β3δ currents, but it failed to potentiate the peak current amplitude and decreased the desensitization of α1β3γ2L currents induced by 1 mM GABA (Figure 2) [26]. In order to determine the structural domains of the δ subunit that are involved in the unique modulation by pentobarbital of α1β3δ currents evoked by a saturating concentration of GABA, a series of chimeras between δ and γ2L subunits were constructed and transfected with wild type α1 and β3 subunits. By comparing the current properties of the chimeric receptors with those of the wild type receptors in the presence of pentobarbital, we concluded that enhancement of α1β3δ currents by pentobarbital required the amino acid sequence from the N terminus to proline 241 in M1 of the δ subunit. We also observed that increasing desensitization of α1β3δ currents by pentobarbital required the amino acid sequence from the N terminus to isoleucine 235 in M1 of the δ subunit [44]. 

Like αβγ receptors, pentobarbital can directly activate αβδ receptors. The peak pentobarbital current amplitude reached its maximal value at 1 mM for α1β3γ2L receptors. However, the peak pentobarbital current amplitude continued to increase up to 3 mM for α1β3δ receptors [26]. It seems that the maximal peak current amplitude evoked by pentobarbital is greater than that evoked by a saturating concentration of GABA for αβδ receptors [26,45]. 

### 3.2. Neurosteroids

The anesthetic action of neurosteroids is achieved by interacting with GABA_A_ receptors [46]. Neurosteroids can either positively or negatively modulate GABA_A_ receptor function. This review focuses on the effect of positive modulators of neurosteroids on αβδ receptor function. Endogenous neurosteroids are produced mainly from glial cells in the brain [40,47], and extracellular concentrations of neurosteroids are in the nanomolar range (10-300 nM), which are dynamically regulated during certain physiological conditions such as pregnancy [10]. Neurosteroids at physiological concentrations predominantly modulate the function of extra- or perisynaptic αβδ receptors [48]. In accord with this, along with the fluctuation of neurosteroid level during pregnancy, the function and expression of αβδ receptors undergo plastic changes in the hippocampus of rodents [49,50,51], which may contribute to alterations of seizure susceptibility and anxiety. It has also been reported that α4βd receptor expression is markedly increased at the onset of puberty in female rodents when some learning processes are impaired [52,53]. Neurosteroids may play an important role in shaping learning deficits at this developmental stage of rodents [53]. 

Neurosteroids such as alphaxalone (5α-pregnan-3α-ol-11,20-di-one) and THDOC (5α-pregnan-3α, 21-diol-20-one) potentiated α4β3δ receptor responses [27,43]. It seems that THDOC evokes a greater enhancement of maximal GABA currents for α1β3δ receptors than for α4β3δ or α6β3δ receptors [24,27,54,55]. THDOC at 1 μM dramatically potentiated the peak current amplitude and increased the desensitization of α1β3δ currents evoked by a saturating concentration of GABA (1 mM). However, THDOC reduced the peak current amplitude and had little effect on desensitization of α1β3γ2L currents evoked by 1 mM GABA. For both receptor isoforms, THDOC prolonged current deactivation [24]. Like pentobarbital, THDOC only slightly enhanced α1β3 currents [24,29], suggesting that the δ subunit plays an important role in modulating α1β3δ currents by these compounds. Interestingly, it was reported that some general anesthetics including THDOC enhanced maximal GABA currents to the similar extent for α4β3δ and α4β3 receptors [55]. These data indicate that it is the α4 subunit instead of the δ subunit that confers the potentiation of α4β3δ currents by THDOC and other anesthetics. Therefore, α subunits may also play a role in modulating αβδ currents by THDOC and general anesthetics. The structural domains of the δ subunit that confer enhancement of αβδ currents by THDOC are not fully elucidated. Preliminary studies suggest that δ subunit domains required for maximal potentiation of α1β3δ currents by neurosteroids may be different from those by barbiturates [44]. 

Application of THDOC (10 nM) suppressed neuronal excitability by enhancing tonic currents mediated by δ subunit-containing receptors in hippocampus [48]. However, for thalamocortical neurons, THDOC at 10 nM had no effect on tonic currents, but application of THDOC at 100 nM did enhance tonic current amplitude [13]. The reasons for this region-specific sensitivity of tonic currents to neurosteroid modulation are currently unclear. Several factors may contribute to this variability. First, neuronal GABA_A_ receptors are highly heterogeneous [14,15]. The composition of αβδ receptors may be different in hippocampal and thalamic neurons. Actually, in thalamus, the major δ subunit-containing receptors are α4βd isoform, and no α1βd isoform is detectable [18,19]. But, both α4βd and α1βd isoforms exist in hippocampus [20,22]. The sensitivity of α1βd and α4βd receptors to the actions of neurosteroids was reported to differ (see above). Second, neurosteroid modulation of GABA_A_ receptor function is dependent on its phosphorylation state. For example, the neurosteroid allopregnanolone prolonged IPSC decay only when the receptors were in a phosphorylated state [56]. Also, PKC activation enhanced the potentiating effect of THDOC on α1β2γ2L currents [57]. Interestingly, the function of α4β3δ receptors was modulated by PKA [58]. Therefore, it is possible that the differential sensitivity of hippocampal and thalamic neurons to neurosteroids is partly due to different phosphorylation levels of αβδ receptors. 

### 3.3. Other Anesthetics

In additional to barbiturates and anesthetic neurosteroids, many other general anesthetics also positively modulate the function of αβδ receptors. For example, etomidate enhances α4β3δ currents [27,55] and augments tonic currents in thalamocortical neurons [12]. Moreover, isoflurane and sevoflurane potentiate recombinant α1β1δ or α6β2δ currents evoked by a sub-maximal concentration of GABA [59,60]. Consistent with these findings, application of isoflurane can lead to enhancement of tonic currents in hippocampal, thalamic and cardiac vagal neurons [61,62,63]. In addition, propofol, a widely used general anesthetic, may also exert its action in the brain partly by modulating tonic inhibition mediated by αβδ receptors. Recent investigations showed that propofol potentiated αβδ currents [25,27,60]. Propofol at 10 μM produced similar alterations for α1β3γ2L and α6β3γ2L currents evoked by a saturating concentration of GABA; peak currents were not changed, desensitization was decreased and deactivation was prolonged [25]. However, propofol at this concentration produced differential effects on α1β3δ and α6β3δ currents. Although propofol potentiated peak current amplitude for both α1β3δ and α6β3δ receptors, the potentiation was greater for α1β3δ than for α6β3δ receptors. Propofol prolonged the deactivation of α6β3δ currents but did not change that of α1β3δ currents. For both receptor isoforms, propofol did not alter the desensitization [25]. In line with these data from recombinant receptors, it has been shown that propofol enhances tonic currents [63,64,65,66,67] and prolongs the duration of miniature IPSCs in neurons [64,67]. Importantly, at clinically relevant concentrations (~0.4 μM) [68], propofol can directly activate α1β3γ2L receptors but produce negligible effect on α1β3δ receptors [25]. Taken together, the evidence suggests that propofol achieves its anesthetic effect in the brain by positively modulating tonic and phasic inhibition as well as by directly activating synaptic GABA_A_ receptors. 

## 4. Modulation of αβδ Receptors by Ethanol

Ethanol is a widely used drug of abuse and exerts its actions in the CNS by interacting with multiple neurotransmission systems including GABAergic transmission [69,70,71]. Previous studies using GABA_A_ receptor δ subunit knockout mice suggest that the δ subunit may play a role in ethanol actions in the CNS [72]. It was subsequently reported that α4βd and α6βd receptors were highly sensitive to ethanol modulation [73,74]. The β3 subunit- other than β2 subunit-containing α4βd and α6βd receptor was considered to be the target for low concentration of ethanol (<30 mM) [74,75]. Ligand binding and electrophysiological studies using alcohol antagonist Ro 15-4513 as a probe indicate that ethanol binds to recombinant and native α4/6β3δ receptors [76,77]. Recent studies investigating the structural domains of the δ subunit have identified key regions of the δ subunit that may be important in conferring ethanol sensitivity in αβδ receptors. It has been reported that loop 2 of the extracellular domain of the GABA_A_ receptor is coupled with channel gating [78], and mutations of the loop 2 residues in the glycine receptor, another member of cys-loop receptors, can affect ethanol sensitivity [79,80]. αβγ receptors are relatively insensitive to ethanol modulation [74]. Interestingly, when loop 2 sequence of the γ2 subunit was replaced with that of the δ subunit, ethanol sensitivity of the chimeric receptor was substantially increased [81], suggesting that loop 2 of the δ subunit plays an important role in conferring high sensitivity of αβδ receptors to ethanol. 

Since the αβδ receptor is the predominant isoform to mediate tonic inhibition and the major contributor to the total GABA_A_ receptor-mediated inhibition in several brain regions [8,13], it is expected that ethanol enhances tonic currents in these areas. In accordance with this, ethanol was reported to potentiate the tonic currents from dentate gyrus granule cells in hippocampus [82,83,84] and thalamocortical neurons in thalamus [85]. In hippocampal interneurons, δ subunits coassemble with α1 subunits to form α1βd receptors, which exhibit high sensitivity to ethanol [22]. Two α6 subunit variants (100R, 100Q) were identified in rat population, and rats carrying the 100Q variant were behaviorally more sensitive to ethanol [87,88,89]. Electrophysiological recordings were performed on cerebellar granule cells in brain slices prepared from rats that were homozygous for either α6 (100R) or α6 (100Q). It was observed that tonic currents were enhanced by ethanol in both genotypes, but the enhancement was greater in slices from the rats with homozygosity of α6 (100Q) [90]. 

It should be noted that not all investigations have been able to confirm the aforementioned findings, resulting in some controversy regarding the ethanol sensitivity of αβδ receptors [75,91,92,93]. For example, several groups failed to observe that ethanol enhanced αβδ currents or bound to αβδ receptors at low concentrations [60,94,95,96]. It was also reported that native αβδ receptors on cultured neurons or on neurons in acute slices were not sensitive to ethanol [60,94,97,98]. A study indicated that the sensitivity of tonic currents to ethanol was not increased for the α6 (100Q) variant in rats [99]. Many confounding factors can lead to the discrepant results such as differences in heterologous expression system, the state of posttranslational modifications of αβδ receptors. It has been recently reported that ethanol enhancement of tonic currents is dependent on the phosphorylation of αβδ receptors mediated by PKCδ [100]. Interestingly, the expression of PKCδ was considerably low in L(tk^−^) cells [100], which were used in a previous study indicating that αβδ receptors were not sensitive to ethanol modulation [94]. This raises a possibility that some of the controversial results may result, at least in part, from the different phosphorylation states of αβδ receptors. That being said, the exact reasons for these discrepancies are largely unknown, and this topic is an area of active research in the alcohol field. 

## 5. Mechanisms for Positive Modulation of αβδ Receptors

Besides anesthetics and ethanol, αβδ receptors can also be positively modulated by many other structurally different compounds such as protons [101,102], gaboxadol (THIP) [43,103], dihydropyrimidinone [104], Tracazolate [105,106] and AA29504 [107]. Although the binding sites for most of these compounds on GABA_A_ receptors have not been identified yet, it seems unlikely that the δ subunit is critically involved in binding of these compounds on αβδ receptors. Previous studies using δ subunit knockout mice suggest that many general anesthetics like barbiturates, etomidate and propofol may not have binding sites on δ subunits [48,108]. Therefore, most anesthetics may modulate αβδ receptor function by modifying the allosteric transduction and gating kinetics of this receptor isoform. 

**Figure 3:**
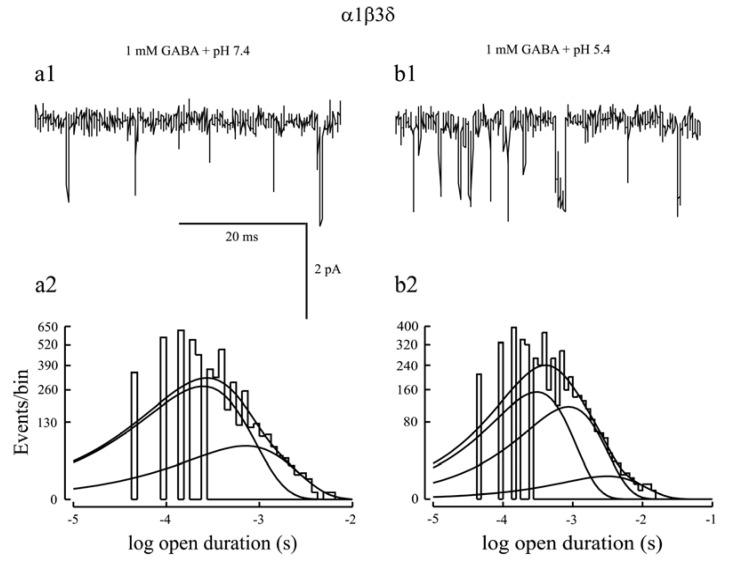
**(a1, b1)** Representative α1β3δ receptor single channel current traces evoked by a saturating concentration of GABA at physiological pH (7.4) and acidic pH (5.4) were presented. The single channel activity at pH 7.4 exhibited brief openings, but that at pH 5.4 displayed a mixture of brief- and long-duration openings; **(a2, b2)** Single channel open probability histograms at physiological pH (7.4) and acidic pH (5.4) were presented. At pH 7.4, the histogram was fitted by two components, indicating two open states. However, at pH 5.4, the histogram was fitted by three components, suggesting that lowered pH introduced an additional open state into α1β3δ receptors to enhance the efficacy of this receptor isoform. Since protons could not directly activate GABA_A_ receptors, the third open state was caused by allosteric modulation of α1β3δ receptors by protons. Modified from [102].

Whole cell currents evoked by saturating concentrations of GABA were consistently smaller for α1β3δ receptors than for α1β3γ2L receptors [24,25,26]. Consistent with this notion, single channel recordings found that α1β3δ channel activity was characterized by brief openings and only had two open states, leading to a shorter mean open time than α1β2/3γ2L receptors [24,26,45,109,110,111]. Additionally, gaboxadol and pentobarbital produced a greater activation than GABA on α1/4β2/3δ receptors [26,43,45]. These aforementioned studies suggest that GABA is a partial agonist for αβδ receptors, leaving much room for potentiation by modulators [112]. Both pentobarbital and THDOC enhanced α1β3δ steady-state current amplitude evoked by a saturating concentration of GABA (see Figure 2a, 2b). Steady-state single channel recordings found that these modulators increased mean open time of α1β3δ currents mainly by introducing a third open state into this receptor isoform [24,26,45]. However, α1β3δ single channel currents activated by pentobarbital (100 μM) or THDOC (1 μM) in the absence of GABA exhibited three open states [24,26]. It is unclear whether the third open state introduced by pentobarbital or THDOC is caused by direct activation or allosteric modulation of α1β3δ receptors. To address this issue, we examined the effect of lowered pH on α1β3δ receptor function since protons allosterically modulate GABA_A_ receptor function without direct activation of it. Lowered pH substantially augmented the steady-state current amplitude of α1β3δ receptors [102]. In line with this, steady-state single channel analysis indicated that at physiological pH, α1β3δ single channel currents were characterized with brief openings and had two open states (Figure 3a1, 3a2) whereas at lowered pH, α1β3δ single channel currents exhibited both brief- and long-duration openings and had three open states (Figure 3b1,b2). These data indicated that lowered pH introduced an additional open state into α1β3δ receptors [102]. Given that the proportion of the third open state in the presence of GABA and pentobarbital (THDOC) is larger than that in the presence of pentobarbital (THDOC) alone, it is conclusive that, like protons, positive modulation of α1β3δ receptors by pentobarbital or THDOC is achieved mainly by introduction of an additional open state. 

Note that pentobarbital also potentiated steady-state current amplitude of α1β3γ2L receptors evoked by a saturating concentration of GABA (see Figure 2c,d). But, pentobarbital achieved this by a different mechanism from that of α1β3δ receptors. Instead of introducing an additional open state, pentobarbital increased steady-state single channel current mean open time by increasing the relative proportion and duration of the third open state for α1β3γ2L receptors [26].

## 6. Conclusions

Although αβδ receptors only constitute a small proportion of all GABA_A_ receptors expressed in the brain, they are the major contributor to GABAergic tonic inhibition in many brain regions. A wealth of studies has demonstrated that tonic inhibition plays an important role in mediating neuronal excitability under physiological conditions. In addition to its involvement in anesthetic and ethanol actions, recent studies showed that defects in αβδ receptor function or tonic inhibition led to pathogenesis of neurological disorders such as epilepsy [31,113,114,115], suggesting that the αβδ receptor is a potential therapeutic target for epilepsy and alcohol abuse. A better understanding of the kinetic properties and mechanisms of allosteric modulation of αβδ receptors should lead to the development of not only highly selective modulators that produce effective anesthesia but also novel treatment strategies to control neurological disorders as well as to reduce the abuse and dependence of alcohol.
